# Supplementary material for: Relationship between Health Counselor Characteristics and Counseling Impact on Individuals at High-Risk for Lifestyle-Related Disease: Sub-Analysis of the J-HARP Cluster-Randomized Controlled Trial
Source: Int J Environ Res Public Health. 2022 May 24;19(11):6375. doi: 10.3390/ijerph19116375 (PMC9179978; doi:10.3390/ijerph19116375)
Supplement: Supplementary file 1 [file ijerph-19-06375-s001.zip › ijerph-1685319-supplementary.pdf]

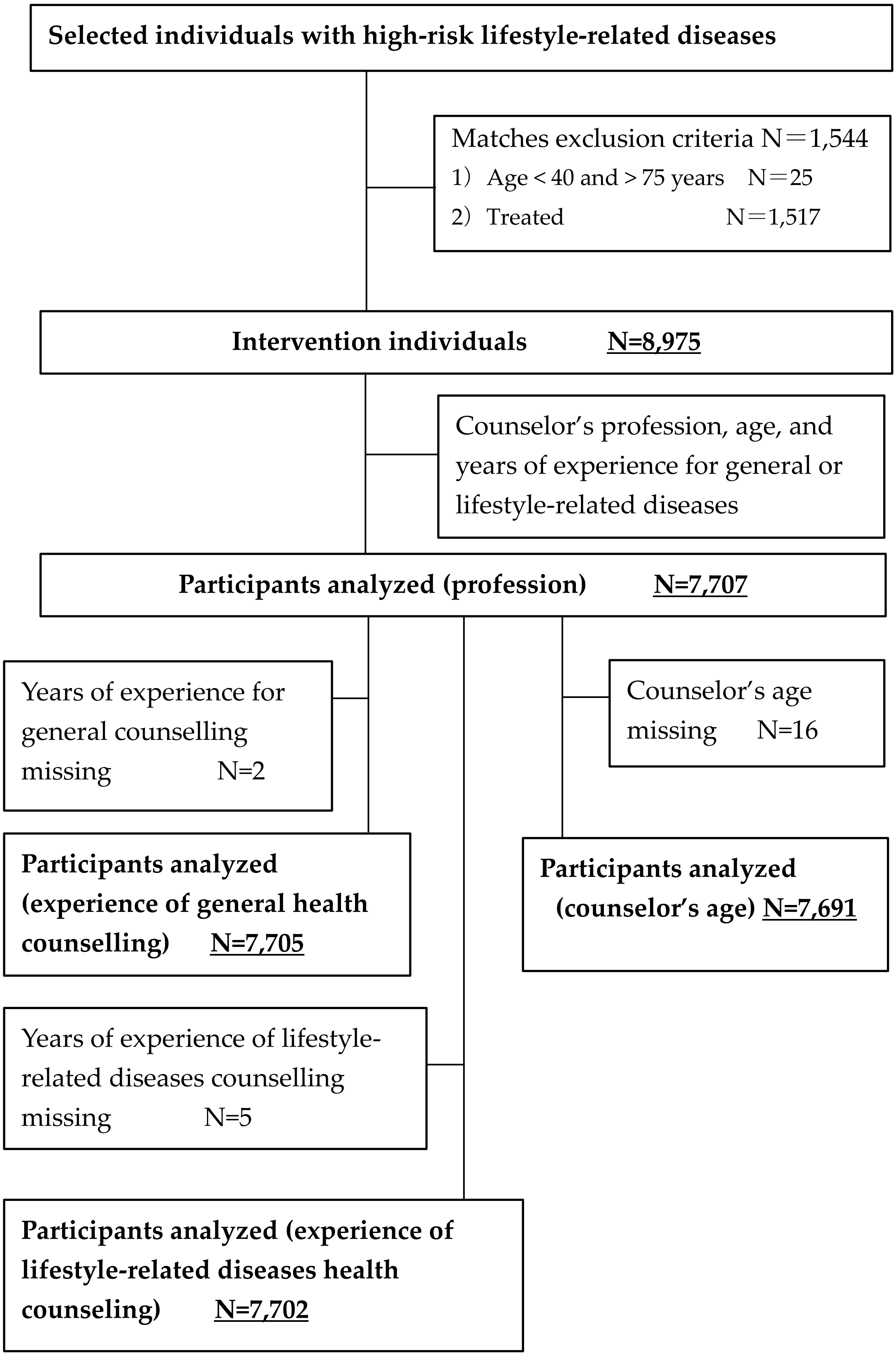

Figure S1. Flow chart of the participants analyzed

Table S1. Characteristics of participants and counselors, and the mode, initial timing, and number of counseling sessions according to years of experience for lifestyle-related disease counseling

Ages of health counselors, years	< 3		3–5		≥ 6	
**Characteristics of participants**						
Number of participants	4,032		1,891		1,779	
Age, years, mean ± SD	63.6	± 8.2	63.0	± 8.7	62.9	± 8.5
Men, n (%)	2,662	(66.0)	1,240	(65.6)	1,170	(65.8)
Grade II or higher hypertension, n (%)	2,376	(58.9)	1,092	(57.8)	1,005	(56.5)
Diabetes mellitus, n (%)	730	(18.1)	371	(19.6)	362	(20.4)
Dyslipidemia among men, n (%)	914	(34.5)	437	(35.4)	386	(33.3)
Proteinuria, n (%)	405	(10.1)	186	(9.9)	198	(11.2)
**Characteristics of counselors**						
Women, n, %	3,961	(98.2)	1,891	(100.0)	1,762	(99.0)
Men, n, %	71	(1.8)	0	(0.0)	17	(1.0)
Profession						
Public health, n (%)	3,418	(84.8)	1,493	(79.0)	1,303	(73.2)
Clinical nurse, n (%)	111	(2.8)	87	(4.6)	398	(22.4)
Nutritionist, n (%)	503	(12.5)	311	(16.5)	78	(4.4)
Years of experience for general counseling, mean ± SD	8.9	± 9.9	12.2	± 9.1	16.3	± 8.3
< 3, n (%)	2,050	(50.9)	5	(0.3)	0	(0.0)
3–9, n (%)	559	(13.9)	1,021	(54.0)	574	(32.3)
10–19, n (%)	674	(16.7)	416	(22.0)	686	(38.6)
≥ 20, n (%)	747	(18.5)	449	(23.7)	519	(29.2)
Counselling mode, n (%)						
Home visit	2,084	(51.7)	994	(52.6)	1,114	(62.6)
Face-to-face in a public place	1,130	(28.0)	522	(27.6)	353	(19.8)
Telephone	192	(4.8)	107	(5.7)	76	(4.3)
Incomplete	626	(15.5)	268	(14.2)	236	(13.3)
Initial timing, n (%)						
≤ 45 days	1,501	(37.2)	743	(39.3)	572	(32.2)
46–90 days	1,077	(26.7)	474	(25.1)	500	(28.1)
≥ 91 days	945	(23.4)	424	(22.4)	492	(27.7)
Incomplete	509	(12.6)	250	(13.2)	215	(12.1)
Number of counseling sessions, n (%)						
1	1,619	(40.2)	831	(44.0)	696	(39.1)
2	1,146	(28.4)	471	(24.9)	561	(31.5)
3	751	(18.6)	377	(19.9)	351	(19.7)
Incomplete	516	(12.8)	212	(11.2)	171	(9.6)

SD: standard deviation

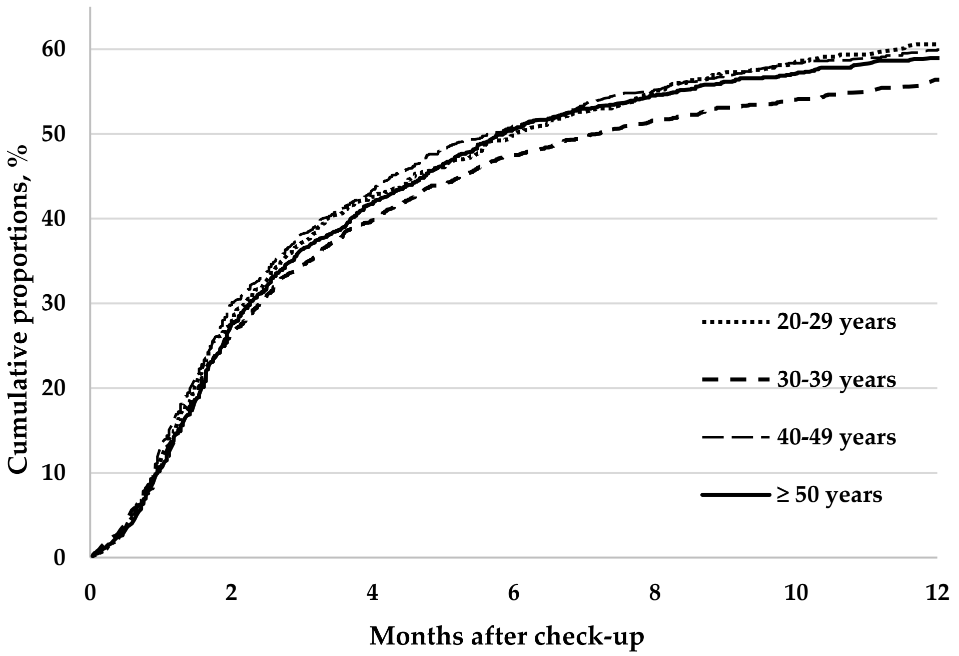

Figure S2. The cumulative proportions of clinical visits for participants according to the health counselor's age

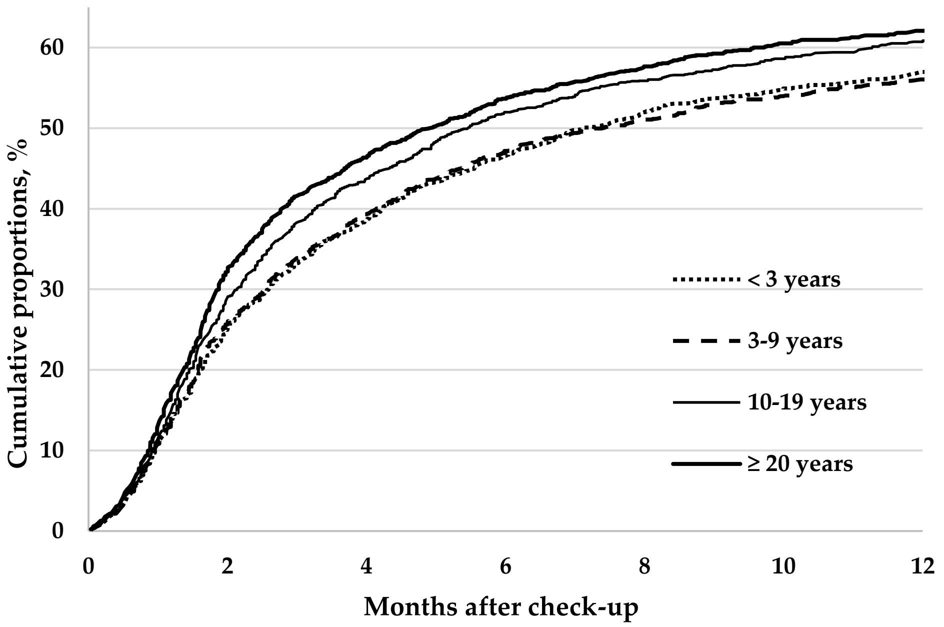

Figure S3. The cumulative proportions of clinical visits for participants according to the years of experience in general counseling

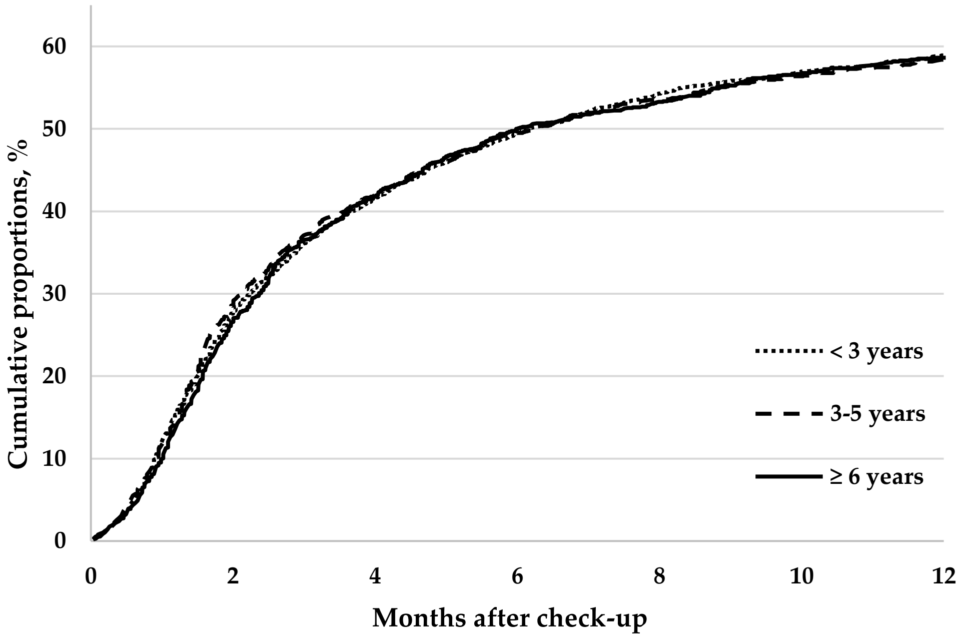

Figure S4. The cumulative proportions of clinical visits for participants according to the years of experience in lifestyle-related disease counseling
